# Isocaloric liquid and solid meals induce comparable postprandial gastric motility: Implications for oral drug delivery assessed by real-time MRI

**DOI:** 10.1016/j.ijpx.2026.100557

**Published:** 2026-05-04

**Authors:** Lydia Neubauer, Simon Bartels, Fiona Mankertz, Dirk Voit, Jens Frahm, Werner Weitschies, Linus Großmann

**Affiliations:** a University of Greifswald, Institute of Pharmacy, Department of Biopharmaceutics and Pharmaceutical Technology, Felix-Hausdorff-Street 3, 17491 Greifswald, Germany; b University Hospital of Tuebingen, Department of Diagnostic and Interventional Radiology, Tuebingen, Germany; c Max Planck Institute for Multidisciplinary Sciences, Biomedical NMR, Göttingen, Germany

**Keywords:** Real-time magnetic resonance imaging, Gastric motility, Gastric emptying, Isocaloric meals, *Magenstrasse*, Postprandial physiology, Artificial intelligence

## Abstract

Postprandial gastric motility critically influences the intragastric behavior of oral dosage forms and subsequent drug absorption. Combining real-time MRI with already established MR imaging, we compared an isocaloric liquid (Fresubin Energy) and a solid meal (egg-white sandwich) in twelve healthy volunteers, assessing gastric emptying, antral contraction frequency, propagation velocity, luminal occlusion, and the intragastric behavior of a non-disintegrating capsule. The water that was ingested together with the capsule, emptied rapidly along the *Magenstrasse* (stomach road), while no meaningful differences in motility parameters were observed between the solid and liquid meals. In this study the capsule remained in the fundus throughout the imaging period, likely due to the combination of fed-state physiology and the supine positioning of the participants. Mean caloric emptying rates were comparable for both meals. These findings suggest that caloric load, rather than physical texture, predominantly governs measurable postprandial motility.

## Graphical Abstract

**Figure:**
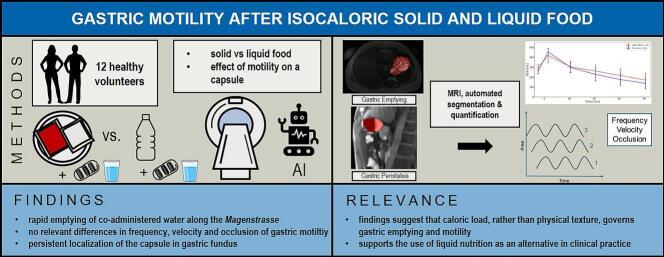

## 1 Introduction

The success of oral drug delivery hinges not only on the formulation itself but critically on the interplay of gastric motor function, intragastric residence, and mechanical processing, which collectively determine the timing and extent of intestinal drug release (Mudie et al., 2010; Dressman and Reppas, 2000). These factors influence drug disintegration and dissolution processes and contribute to the variability observed in systemic exposure following oral administration under fed conditions (Koziolek et al., 2018).

A fundamental distinction in postprandial gastric physiology must be made between non-caloric liquids (e.g. water) and caloric meals. Water empties rapidly from the stomach via the so-called *Magenstrasse*, largely bypassing extensive antral processing and exhibiting first-order flow-dependent emptying kinetics (Grimm et al., 2018; Grimm et al., 2017). In contrast, both caloric liquid meals and solid meals are emptied under feedback control governed primarily by nutrient sensing in the proximal small intestine, resulting in regulated, calorie-dependent emptying. While solid meals additionally require mechanical breakdown by coordinated antral peristalsis before particles can pass the pylorus, overall gastric emptying of caloric content is constrained by caloric delivery rather than physical state alone (Calbet and MacLean, 1997; Camilleri, 2019; Goyal et al., 2019; Velchik et al., 1989).

From a pharmaceutical perspective, the question of whether meal consistency per se, liquid versus solid, modulates gastric motility in a manner relevant for drug absorption, remains incompletely resolved. Patients may ingest medications with liquid nutritional supplements, semi-solid foods, or solid meals, whereas fed-state pharmacokinetic (PK) studies typically rely on standardized solid meals without considering alternative caloric textures (FDA, CDER, 2002; EMA, 2010). If calorically matched liquid and solid meals induce different intragastric motor patterns, such as differences in contraction frequency, propagation velocity, or luminal occlusion, this might alter mechanical stress exposure of dosage forms, intragastric residence behavior, and the timing of intestinal drug delivery. Conversely, if gastric motor patterns are comparable under controlled caloric conditions, the pharmacokinetic relevance of meal texture alone may be limited.

The existing literature provides conflicting evidence. Some human crossover studies report similar gastric emptying profiles for isocaloric liquid and solid meals, suggesting that energy content is the dominant determinant of postprandial gastric processing (Achour et al., 2001; Van Natta et al., 2022; Solnes et al., 2019; Sachdeva et al., 2013). Other studies describe differences in emptying dynamics or early postprandial behavior, particularly related to lag phases or temporal patterns, indicating that meal structure may still influence gastric motor function in ways not fully captured by global emptying metrics (Achour et al., 2001; Kunz et al., 1999; Marciani et al., 2001a). Importantly, many of these investigations rely on scintigraphic or indirect techniques with limited temporal and spatial resolution, restricting detailed analysis of dynamic motility parameters (Abell et al., 2008; Urbain and David Charkes, 1995; de Jonge et al., 2018).

In parallel, pharmaceutical research has demonstrated that gastric motility can directly influence the in vivo behavior of oral dosage forms. Postprandial contraction patterns and intragastric pressure profiles have been shown to affect disintegration, drug release, and intragastric retention of dosage forms, particularly for capsules, modified-release formulations, and gastroretentive systems (Bardonnet et al., 2006; Omidian, 2025; Schneider et al., 2018). These findings underscore that gastric emptying time alone is insufficient to describe the mechanical environment relevant for oral drug performance; rather, detailed characterization of gastric motor patterns is required.

Recent advances in real-time magnetic resonance imaging (rtMRI) now enable radiation-free, high-temporal-resolution visualization of gastric motility in vivo. In our previously published work, we firstly described a quantitative rtMRI framework for assessing gastric motor function under standardized conditions (Neubauer et al., 2026). This approach allows objective measurement of motility parameters such as contraction frequency, propagation velocity, and luminal occlusion degree, thereby providing a level of mechanistic and spatial detail that was not achievable with earlier methodologies.

Building on this new imaging platform, the present study aimed to systematically investigate the influence of meal consistency on gastric motility under isocaloric conditions, focusing on parameters directly relevant to oral drug delivery. In addition, the intragastric behavior of an ingested enteric capsule was assessed to explore potential interactions between postprandial motor activity and luminal object dynamics. By combining contemporary imaging precision with a pharmaceutically motivated research question, this work seeks to refine the physiological basis underlying fed-state variability in oral drug delivery.

## 2 Materials and Methods

### 2.1 Participants

The present study represents an amendment to a previously published protocol (Neubauer et al., 2026). This amendment study protocol was approved by the Ethics Committee of the University Medicine of Greifswald (BB072/24a, DRKS00035519). The study was conducted in accordance with the latest version of the Declaration of Helsinki. All participants provided written informed consent.

12 healthy participants were recruited. Inclusion criteria were age between 18 and 55 yrs., BMI between 18 kg/m^2^–30 kg/m^2^ and health, assessed by the investigating physician as clinically unremarkable. Exclusion criteria were any absolute or relative contraindications to MRI.

### 2.2 Study Protocol

The two-arm, open-label, non-randomized study amendment was conducted at the Department of Diagnostic Radiology and Neuroradiology at the University Medicine Greifswald. Alcohol consumption had to be stopped at least 24 h before the study day. The subjects were not allowed to eat for at least 10 h, and caloric drinks were to be avoided for 3 h and water for 90 min prior to the study. The consumption of caffeinated beverages was not permitted.

**Fig. 1:**
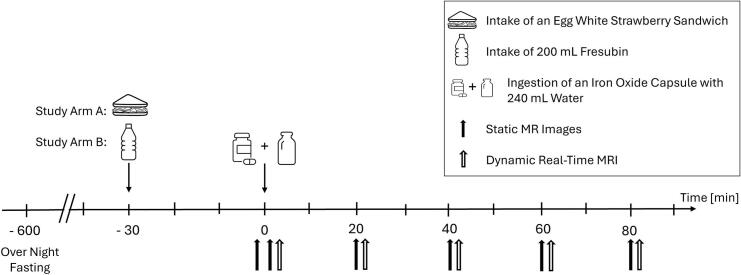
Study Procedure: Timeline with Interventions Explained in Legend.

All subjects participated in both study arms. As can be seen in Fig. 1, subjects consumed an egg-white strawberry sandwich (referred to below as light meal) in study arm A and 200 mL Fresubin Energy in study arm B within 15 min at *t* = − 30 min. 25 min later subjects underwent MRI for a reference measurement at t = − 3 min. Then, subjects ingested an enteric capsule with 240 mL water in an upright position at *t* = 0 min. Within this protocol, two complementary MRI acquisition types were applied. At time points *t* = 2, 20, 40, 60, 80 min, static MR images (Volumetric Interpolated Breathold Examination (VIBE)) were acquired for gastric content volume assessment. Dynamic rtMRI were acquired at *t* = 4, 22, 42, 62, 82 min for the evaluation of gastric motility. Participants remained in the supine position within the MRI scanner for the entire duration of the imaging period and did not leave the scanner between measurements.

### 2.3 Composition of the Meals

To compare solid with liquid food, the egg-white strawberry sandwich was used as solid meal as it is routinely used as standard meal for the scintigraphic determination of gastric emptying and Fresubin Energy (Fresubin Energy Drink, Fresenius Kabi, Kriens, Switzerland) as a typical liquid meal were used. The standard meal is recommended by the *American Neurogastroenterology and Motility Society* and the *Society of Nuclear Medicine* and consists of 118 mL egg-whites (Pumperlgsund Freilande, Pumperlgsund GmbH, Munich, Germany), two slices of toasted white bread (Sammy's Super Sandwich, Harry-Brot GmbH, Schenefeld, Germany) and 30 g strawberry jam (Erdbeerkonfitüre Extra Gut & Günstig, EDEKA ZENTRALE Handels Stiftung & Co. KG, Hamburg, Germany) (Donohoe et al., 2009). This meal contains 255 kcal. To maintain the identical calorie content as Fresubin Energy (200 mL, 300 kcal), 6 g of butter (Meggle Alpenbutter, MEGGLE GmbH & Co. KG, Wasserburg, Germany) were added to the sandwich. For comparison, Table 1 shows the nutritional composition of both meals.

**Table 1:** Nutritional Composition of the Light Meal and Fresubin Energy.

Nutritional Composition (%)	Light Meal	Fresubin Energy
Carbohydrates	57	50
Proteins	28	15
Fats	13	35
Dietary Fibers	2	0

The food additive manganese(II) gluconate dihydrate (Dr. Paul Lohmann GmbH und Co., Emmert, Germany) was used for T1-contrast. 168.2 mg were added to Fresubin Energy, which already contains 0.8 mg manganese (as Mn^2+^), and was homogenized right before the consumption. At the time of application, Fresubin Energy had a temperature of approximately 15 °C. 175.2 mg manganese(II) gluconate dihydrate were added to the egg-white and homogenized before it was cooked in the microwave at 800 W for 2 min. Both meals contained 20 mg manganese (as Mn^2+^).

### 2.4 Oral Dosage Form

First, 200 g of a powder mixture for tableting was prepared for the oral dosage form. This consisted of 97% anhydrous calcium hydrogen phosphate (JRS Pharma GmbH & Co. KG, Rosenberg, Germany), 1% highly dispersed silica (type 200) (Caesar & Loretz GmbH, Hilden, Germany) and 2% magnesium stearate (Parmentier & Co GmbH, Frankfurt a.M., Germany). The calcium hydrogen phosphate was weighed, and the corresponding amount of silicon dioxide was sieved through a 500 μm sieve. This was then mixed with the calcium hydrogen phosphate for 5 min at 49 rpm in the TURBULA® mixer (Willy A. Bachofen AG, Muttenz, Switzerland). Then, the corresponding amount of magnesium stearate was sieved through a 500 μm sieve and mixed with the powder mixture again in the TURBULA® mixer for 1 min. Finally, 0.5% black iron(II,III) oxide (Caesar & Loretz GmbH, Hilden, Germany) was added to the mixture for contrast and mixed proportionally. The tablets were manufactured using an eccentric mechanical press (EK 0 Korsch, Nagema VEB Maschinen und Mühlenbau, company closed). A biplane punch set with a diameter of 6 mm was selected. Tablets were compressed, resulting in a breaking strength of circa 155 N, and weighed circa 300 mg each. Enteric-coated Capsugel® Enprotect® size 0 capsules (Lonza Group AG, Basel, Switzerland), which are based on HPMC and HPMC-AS were used for packaging of the tablets. A previous study showed that the capsules are stable in the stomach under postprandial conditions and only disintegrate in the small intestine after gastric emptying (Grimm et al., 2023). Each capsule was filled with three tablets to achieve a calculated density of 1.43 g/cm^3^ (the total mass of the dosage form was approximately 1 g, considering the internal volume of a size 0 capsule, which is approximately 0.70 cm^3^).

### 2.5 MRI Acquisition

MRI were acquired on a clinical 3 T MRI scanner (MAGNETOM Vida, Siemens Healthineers, Germany) with an 18-channel body and 72-channel spine coil in supine position. T1-weighted spoiled GRE sequences (VIBE) were used to visualize the abdomen in the coronal and transverse planes. Transversal images were used to determine the gastric content volume (GCV) over time. The coronal VIBE protocol used a TR = 5.99 ms, TE = 2.46 ms, slice thickness of 4 mm, 60 slices, flip angle = 30°, matrix = 320 × 161, FOV = 550 × 395 mm and a 20 s inspiratory breath-hold. Transverse VIBE used matched parameters but with TR = 6.03 ms, matrix = 448 × 176 and FOV = 550 × 309 mm. To analyze peristalsis, T1-weighted radial real-time spoiled GRE sequences (FLASH2) were acquired in sagittal plane (Uecker et al., 2010). This sequence used a TR = 3.08 ms and TE = 1.9 ms, slice thickness of 5 mm, and three interleaved slices with a flip angle of 20°, matrix = 140 × 140, and FOV = 200 × 200 mm. Each frame consisted of 13 radial spokes, resulting in an effective frame rate of 6.24 fps including the time for CHESS fat saturation before each frame. FLASH2 data were acquired for 180 s during free breathing. FLASH2 slice positioning was guided by transverse VIBE images (at *t* = 2 min) and kept constant. Slices were positioned relative to the antrum/pylorus: slice 1 anterior to the pylorus, slice 2 mid-antrum, slice 3 near the lesser curvature. The distance factor (DF) between the slices was adapted individually (1.5–3.0), yielding inter-slice distances of 7.5–15 mm.

### 2.6 Artificial Intelligence (AI)

Gastric segmentation used two already trained nnU-Net models: *Volyntra* for transverse volumetry and *Motiqva* for sagittal real-time sequences to extract peristalsis from area-time curves (Isensee et al., 2021). To ensure that both AIs could recognize and segment the new stomach contents (sandwich and Fresubin Energy), the existing AIs were retrained with new data from this study. For this purpose, three participants data sets per study arm were manually segmented and used for retraining (manual segmentation: S.B. 0.5 yrs. expertise, review and editing: L.N. 2 yrs. expertise, L.G. 5 yrs. expertise in gastrointestinal imaging). How the segmentations, retraining of the AIs and the inference were carried out is explained in detail elsewhere (Neubauer et al., 2026). Importantly after segmentation for each participant and time point .mp4 videos showing the MR sequence overlaid with the generated segmentation masks were generated. This video output enabled the inspection of the AI segmentation quality in a human-in-the-loop process. For this all videos were inspected (S.B., L.N., L.G.) and in case of a false segmentation, the sequence plus matching label file were loaded into 3D Slicer (version 5.8.1, The Slicer Community, USA) for manual correction (Fedorov et al., 2012). Manual correction of AI-based segmentations was performed when predefined quality criteria were not met. For *Volyntra*, this included cases of incomplete segmentation (missing parts of the gastric contents) or over-segmentation (inclusion of regions outside the gastric contents). For *Motiqva*, corrections were applied when segmentations extended beyond the stomach lumen (including stomach contents and intragastric air) or when the stomach was only partially segmented. All segmentations were visually inspected, and corrections were performed as needed to ensure anatomical plausibility and consistency across time points. After editing, the corrected label file was reintegrated into the analysis of volume and peristalsis.

### 2.7 Volume Evaluation

Gastric content volume (GCV) was computed by voxel counting in the segmented transverse VIBE masks and conversion to mL using voxel dimensions. Emptying rates were calculated as the linear slope of the decrease in gastric content volume over time between 41 and 81 min, where the emptying rate = ΔV/Δt. This time period was chosen because it can be assumed that the added water had already been emptied at the 41-min mark. To calculate the emptying rate in calories per minute, the volume value at *t* = −5 min was first set to 300 kcal. We are aware that this value is not physiologically accurate, as digestion will have begun by this point, and the volume will consist of secretions and water. However, this value provides a rough estimate. Here, too, the period between 41 and 81 min was selected, the corresponding volume values were converted from the reference value in kilocalories, and the emptying rate in kcal/min was calculated using Δkcal/Δt.

GCVs were baseline-corrected to the initial post-ingestion measurement to isolate volume changes attributable to gastric emptying of the water and test meals. ΔGCV was calculated as: ΔGCV(t) = GCV(t) - GCV(−5 min).

### 2.8 Motility Evaluation

For motility evaluation the corresponding frequencies, velocities and occlusions were extracted from segmented area-time curves obtained from sagittal FLASH2 sequences. Values were calculated as in the published methodological paper. Shortly, dominant contraction frequencies were identified via Fast Fourier Transformation (FFT) within the band of 0.03–0.07 Hz (1.8–4.2 contractions per minute (cpm)). Frequencies with a dominance factor ≥ 2.0, defined as the ratio of peak amplitude to the mean amplitude within the band, were considered valid indicators of peristaltic activity. Mean values for wave frequency and velocity (calculation based on frequency) were calculated only at time points in which at least two out of three sagittal slices exhibited a dominance factor ≥ 2.0. To calculate the velocity, the required propagation distance was needed, which was calculated as 3 times the slice thickness added to 2 times the individual inter-slice distance. To obtain a spatio-temporal metric, an apparent propagation speed was computed as (Eq. 1):

$$\mathrm{Eq}.1:\mathrm{Apparent Propagation Speed}\;\left[\frac{\mathrm{mm}}{s}\right]=\left(\frac{\mathrm{Frequency}\;\left[\mathrm{cpm}\right]}{60}\right)\times \mathrm{Propagation Distance}\;\left[\mathrm{mm}\right]$$

Occlusion can be interpreted as an imaging-derived surrogate of contraction amplitude, representing the spatial extent of luminal narrowing during gastric contractions. In our study occlusion was defined as the relative reduction in cross-sectional area during a contraction, normalized to a reference area. For every subject, both study arms, each measurement time point of the study protocol and for each of the three sagittal slices, all frames of the corresponding real-time sequence were analyzed separately. The reference area was calculated per slice and per measurement time point as the median of the top 10% largest areas within this sequence. Occlusion was calculated as (Eq. 2):

$$\mathrm{Eq}.2:\mathrm{Occlusion}\;\left[\%\right]=100\times \left(1-\frac{\mathrm{Area}}{\mathrm{Median of}\;\mathrm{top}\;10\%\mathrm{Areas}}\right)$$

with all negative values clipped to 0%, as occlusion cannot be smaller than the fully relaxed state. Mean occlusion values were computed per time point and slice to estimate contractile strength, with complete antral closure corresponding to 100% occlusion.

### 2.9 Statistics

Statistical analyses were performed using *jamovi* (version 2.6 The jamovi project, Australia) with the GAMLj module (https://www.jamovi.org/, n.d.). Linear mixed-effects models (LMMs) were used for all outcome variables to account for repeated measurements within subjects. Model complexity was kept intentionally parsimonious given the sample size, and higher-order interaction terms were only interpreted if supported by statistical evidence.

Depending on the parameter, models included the fixed effects *timepoint, product* (Fresubin Energy vs. egg-white sandwich), and, where applicable, *slice number*, as well as all relevant two- and three-way interactions. *Slice number* was included as a fixed effect in the statistical models as a spatial surrogate for anatomical position along the gastric axis, representing a proximal-to-distal gradient from fundus/antrum towards the pylorus. This approach allowed the assessment of region-specific differences in gastric motility and occlusion patterns within the stomach.

All models included a random intercept for subject. Degrees of freedom were estimated using the Satterthwaite approximation. Residual normality was assessed using Kolmogorov–Smirnov and Shapiro–Wilk tests, supported by visual inspection of Q–Q plots. Statistical significance was defined as *p* < 0.05.

## 3 Results

Twelve healthy participants (6 female, mean 25 ± 4 yrs., BMI 23.4 ± 2.0 kg/m^2^) were included and completed the prospective study, with no reports of adverse events.

### 3.1 Human-in-the-Loop: Manual Correction

Of a total of 144 VIBE sequences (6 time points, 12 subjects in two study arms) for gastric emptying analysis, 17 (11.8%) sequences from 5 subjects in study arm A and 3 subjects in study arm B had to be corrected manually. For peristalsis from a total of 360 rtMR sequences (5 time points, 3 slices, 12 subjects in two study arms), 59 (16.4%) sequences from 8 subjects in study arm A and 9 subjects in study arm B had to be corrected manually. Like in our first publication of this method, segmentation performance was higher for *Volyntra* than for *Motiqva.* The sequences to be corrected were always individual frames, not the entire sequence.

### 3.2 Gastric Content volume

**Fig. 2:**
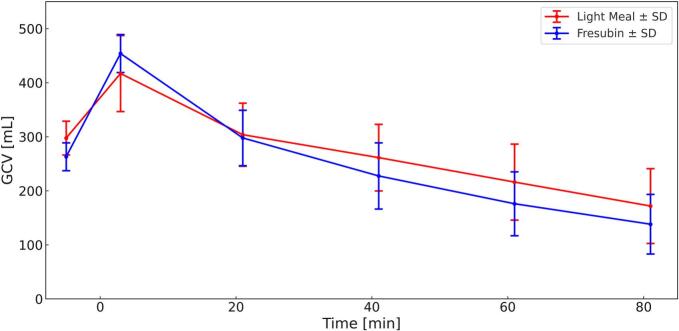
Gastric Content Volume (GCV) for both Meals (Light Meal = Red, Fresubin Energy = Blue) over 82 min. *N* = 12, Mean ± SD. (For interpretation of the references to colour in this figure legend, the reader is referred to the web version of this article.)

Fig. 2 illustrates the mean gastric content volume (GCV) over time for both meals. At baseline (*t* = 0), ingestion of water resulted in a marked increase in GCV in both study arms (mean: light meal: 417 ± 70 mL, Fresubin Energy: 454 ± 35 mL). The light meal exhibited a higher initial mean gastric volume of 298 ± 31 mL, whereas Fresubin Energy with 263 ± 26 mL showed a mean volume that was 35 mL lower.

**Fig. 3:**
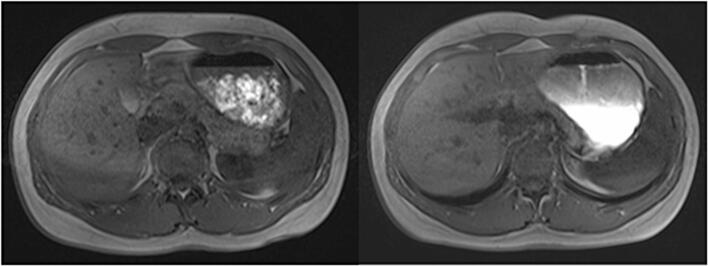
Comparison of the Stratification of the Consumed Water in Participant P003 in Transverse VIBE at *t* = 3 min, Left: Light Meal, Right: Fresubin Energy.

Visual inspection suggested that the ingested water tended to layer above Fresubin Energy, whereas more apparent mixing was observed with the light meal (Fig. 3). However, this observation was not quantitatively assessed and should therefore be interpreted cautiously. During the first 20 min, a pronounced and rapid decline in GCV was observed in both arms, indicating fast emptying of the ingested water. Thereafter, gastric volume decreased more gradually and remained relatively stable over the remaining observation period.

**Fig. 4:**
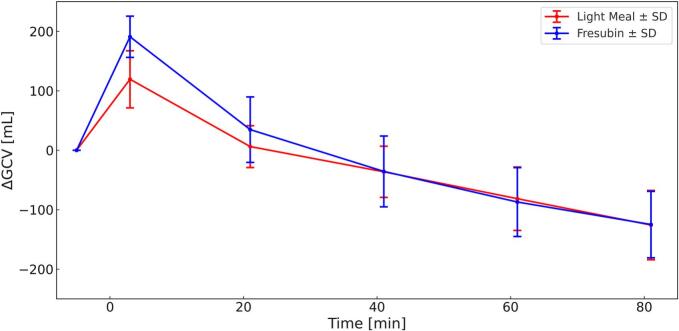
ΔGCV for both Meals (Light Meal = Red, Fresubin Energy = Blue) over 82 min. *N* = 12 Mean, ± SD. (For interpretation of the references to colour in this figure legend, the reader is referred to the web version of this article.)

Fig. 4 presents the change in GCV (ΔGCV) relative to *t* = −5 min (volume of the meal alone) and demonstrates a clear biphasic emptying pattern, characterized by an initial rapid emptying phase attributable to water, followed by a slower, nearly linear emptying of the caloric meals. After completion of the initial water emptying phase (*t* = 41 min), gastric emptying rates differed minimally between the two meals. The emptying rate of the light meal was 2.24 **±** 0.65 mL/min, corresponding to an energy emptying rate of 2.26 **±** 0.61 kcal/min, whereas Fresubin Energy emptied at 2.23 **±** 0.58 mL/min, equivalent to 2.55 **±** 0.65 kcal/min.

Linear mixed-effects modeling revealed significant main effects of *time* and *product* as well as a significant *time × product* interaction.

Post-hoc analysis showed that gastric content volume differed significantly between the two meals only during the early phase between *t* = −5 and *t* = 3 min, with Fresubin Energy exhibiting a higher volume compared to the light meal.

No significant differences in GCV (all *p* > 0.16) or ΔGCV (all *p* > 0.12) were observed at any subsequent time points.

### 3.3 Gastric Motility

**Fig. 5:**
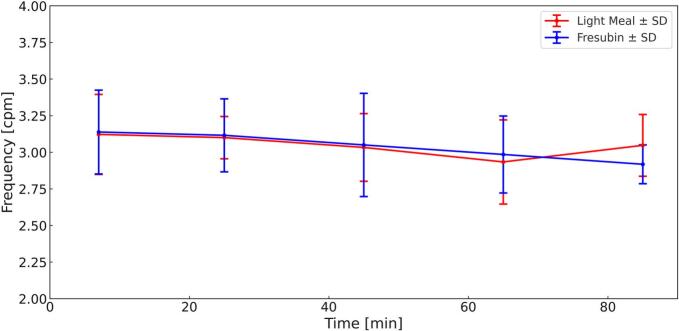
Mean Frequency ± SD (Light Meal = Red, Fresubin Energy = Blue) over 85 min. (For interpretation of the references to colour in this figure legend, the reader is referred to the web version of this article.)

Gastric peristaltic wave frequency remained stable over time in both study arms (Fig. 5). Following ingestion of the light meal, mean wave frequency ranged from 3.12 ± 0.27 cpm at 7 min to 3.05 ± 0.21 cpm at 85 min. Similarly, Fresubin Energy exhibited comparable frequencies throughout the observation period, decreasing slightly from 3.14 ± 0.29 cpm at 7 min to 2.92 ± 0.13 cpm at 85 min. At all measured time points, wave frequency was similar between the two meals, with values consistently close to 3 cpm.

Statistically a significant main effect of *time* was observed, indicating a modest decrease in wave frequency over the postprandial period. In contrast, no significant effect of *meal* (*p* = 0.168) was observed.

**Fig. 6:**
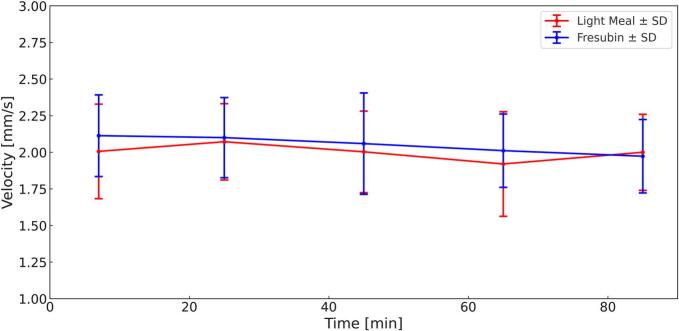
Mean Velocity ± SD (Light Meal = Red, Fresubin Energy = Blue) over 85 min. (For interpretation of the references to colour in this figure legend, the reader is referred to the web version of this article.)

The mean propagation velocity of gastric peristaltic waves showed only minor temporal variation in both groups (Fig. 6). For the light meal, velocities ranged between 2.01 ± 0.32 mm/s at 7 min and 2.00 ± 0.26 mm/s at 85 min, with a slight decrease observed at later time points.

After ingestion of Fresubin Energy, wave velocities were higher (2.11 ± 0.28 mm/s at 7 min) and gradually decreased to 1.97 ± 0.25 mm/s at 85 min, reflecting a gradual reduction in propagation velocity over time.

A significant main effect of *meal* was identified, with Fresubin Energy exhibiting overall higher wave velocities compared to the light meal. No interaction effects between *time*, *meal*, and *slice* were detected (all *p* > 0.73).

Although some temporal effects reached statistical significance in terms of frequency and velocity, the observed changes were small in magnitude and are consistent with expected physiological variability in the postprandial state rather than indicating major alterations in gastric motor functions.

**Fig. 7:**
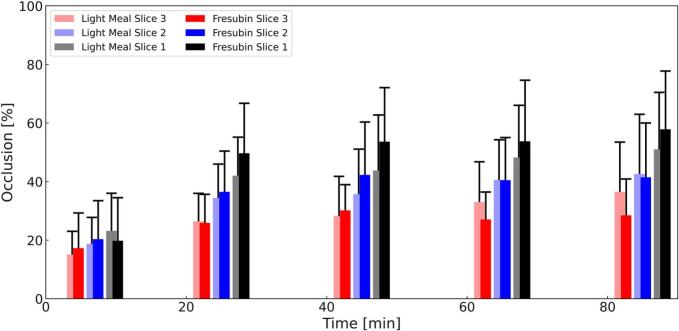
Mean Occlusion ± SD (Light Meal = Red, Fresubin Energy = Blue) over 85 min. *N* = 12. (For interpretation of the references to colour in this figure legend, the reader is referred to the web version of this article.)

Occlusion increased progressively over time in both study arms and across all analyzed three slices (Fig. 7). Following ingestion of the light meal, mean occlusion in slice 1 increased from 23.2 ± 12.9% at 7 min to 51.0 ± 19.5% at 85 min. Fresubin Energy showed slightly higher occlusion values at corresponding time points, increasing from 19.8 ± 14.7% at 7 min to 57.8 ± 19.9% at 85 min.

A significant main effect of *time* was observed, indicating a progressive increase in occlusion over the postprandial period. In addition, a significant effect of *slice* was detected, with occlusion consistently highest in slice 1, followed by slice 2, and lowest in slice 3, so increasing towards the pylorus.

No significant effect of *meal* was observed, and no interaction effects between *time* and *meal* or between *meal* and *slice* were detected, indicating comparable occlusion patterns for the light meal and Fresubin Energy across all time points.

The numbers of participants included in each timepoint for the calculation of frequency and velocity are reported in Table 2. In general, regular peristalsis was observed in most cases. However, for Fresubin Energy, the N-number is higher than for the light meal, with e.g. 10 evaluable data sets at the last time point *t* = 85 min compared to 7 data sets for the light meal.

**Table 2:** Number of Participants included for the Calculations of Frequency and Velocity for all Measurement Timepoints.

Time (min)	Number of Participants (Light Meal)	Number of Participants (Fresubin Energy)
7	10	12
25	11	12
45	10	11
65	10	10
85	7	10

### 3.4 Capsule

**Fig. 8:**
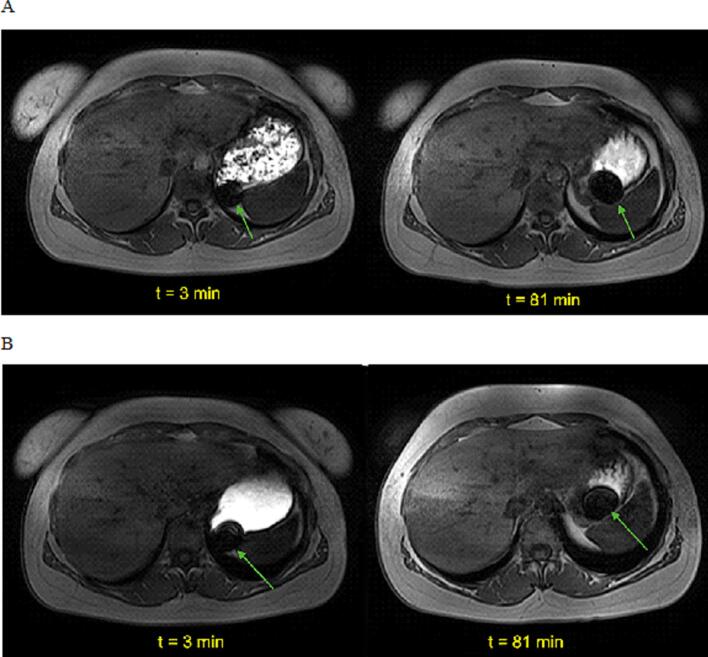
Iron(II,III) Oxide Susceptibility Artifacts (Green Arrows) in Transverse VIBE at 3 and 81 min for Participant P001 after Consumption of A: Light Meal, B: Fresubin Energy. (For interpretation of the references to colour in this figure legend, the reader is referred to the web version of this article.)

In the MR images, the enteric-coated capsule filled with three iron oxide tablets, which was ingested at *t* = 0 min, was clearly visible as a black susceptibility artifact in all participants. The capsule remained in the fundus of the stomach in all subjects throughout the entire examination period. Due to its position, the susceptibility artifact of the capsule was not visible in the sagittal rtMR videos but was detectable in the fundus at all time points in the VIBE sequences. These results were identical for both study arms. No significant change in intra-gastric positioning was observed over time or depending on the type of food. In Fig. 8, the susceptibility artifacts, exemplary for one subject and both study arms, are highlighted. Furthermore, no fragmentation or release of the capsule contents was observed during the observation period in any cases.

## 4 Discussion

In this study, we compared an isocaloric liquid and a solid meal under controlled conditions and assessed their impact on gastric motor parameters and capsule behavior using rtMRI. Three main findings emerged: rapid emptying of co-administered water consistent with preferential flow along the *Magenstrasse*, no meaningful differences between the isocaloric liquid and solid meals in contraction frequency, apparent propagation velocity, or luminal occlusion, and persistent localization of the capsule in the gastric fundus without observable emptying during the study period.

The rapid emptying of water in the first 20 min is consistent with several descriptions of the preferred transport of fluid along the *Magenstrasse* in the stomach, as already described in studies by our working group (Grimm et al., 2017; Großmann et al., 2024; Großmann et al., 2025). In contrast, both caloric test meals, despite differences in physical structure, appeared to be governed by similar postprandial motor organization, consistent with evidence that gastric emptying is regulated to maintain a relatively constant caloric delivery to the small intestine through nutrient-dependent feedback mechanisms (Beglinger and Degen, 2006; Feinle-Bisset and Horowitz, 2021; Maljaars et al., 2007; Pilichiewicz et al., 2007; Marathe et al., 2013). The choice of the scintigraphic standard meal (egg-white strawberry jam sandwich), adjusted by the addition of a small amount of butter to match the caloric content of Fresubin Energy, was intentional. This meal is widely used in gastric emptying scintigraphy and serves as an established reference standard in gastric emptying diagnostics. By selecting this established solid test meal and adapting it to achieve isocaloric conditions, we aimed to ensure comparability with scintigraphic literature while enabling a controlled assessment of meal consistency effects within the MRI setting. The design therefore allows a methodological bridge between established nuclear medicine protocols and rtMRI-based motility analysis. Although both meals were isocaloric, their macronutrient composition differed. Lipids are known to exert the most potent inhibitory effects on antropyloroduodenal motility and gastric emptying, primarily via CCK-mediated feedback mechanisms, followed by protein and carbohydrates (Pilichiewicz et al., 2007; Weigle et al., 2012; Feinle et al., 2003; Little et al., 2007). However, the references listed also emphasize that the difference in nutrients must be highly significant in order to achieve an effect and it should also be noted that some of the relevant components were infused directly into the duodenum.

Underlying this, no relevant differences were observed in antral contraction frequency or luminal occlusion between the solid and liquid isocaloric conditions. For propagation velocity, a statistically significant main effect of meal was identified, with slightly higher values in the Fresubin Energy condition. However, the absolute difference was small (mean difference: 0.5 mm/s) and, like the contraction frequency (≈ 3 cpm), the velocity remained within the physiological ranges reported for healthy adults under fed conditions (≈ 2 mm/s) (Marciani et al., 2001b; O'Grady et al., 2022), further supporting the validity of our rtMRI approach. In addition, no interaction effects were detected, and no consistent meal-related differences were observed across other motility parameters. Therefore, this finding is not interpreted as evidence of a relevant alteration in overall gastric motor function but rather as a subtle variation within physiological variability.

Although numerous studies report distinct emptying kinetics for solids and liquids, these differences typically relate to the presence of an initial lag phase for solids, particle trituration, or differences in overall emptying half-times rather than to fundamental alterations in the underlying contractile rhythm (Goyal et al., 2019; Marciani et al., 2001a). In our study, the initial lag phase of the solid meal could not be systematically assessed, as imaging commenced after meal ingestion in accordance with a standardized postprandial protocol. This approach was chosen to align with typical pharmacokinetic study designs, in which drug administration and subsequent assessments occur after a defined waiting period of 30 min following meal intake. Consequently, our analysis primarily reflects established postprandial motor activity rather than the very early phase of solid gastric accommodation and trituration.

Experimental evidence has nevertheless long demonstrated that gastric emptying rates are closely linked to caloric density and small-intestinal nutrient exposure, largely independent of meal volume once a caloric threshold is reached (Hunt and Stubbs, 1975). This caloric regulation is mediated via duodenal chemosensing and the release of inhibitory gut peptides such as cholecystokinin and glucagon-like peptide-1, which modulate antral and pyloric motor function (Hellström et al., 2006; Holst, 2007). Contemporary reviews further emphasize that, in the fed state, interdigestive motility is suppressed and replaced by a coordinated postprandial motor pattern governed predominantly by nutrient feedback mechanisms rather than by physical consistency per se (Deloose et al., 2012; Janssen et al., 2011; Camilleri et al., 1985). Within this regulatory framework, isocaloric liquid and solid meals, despite differing nutrients, are expected to evoke comparable motor organization once intragastric homogenization (mixing, dilution by secretion, emulsification) has occurred. Consistent with this, the measured mean caloric emptying rates were rather similar for the solid meal (2.26 kcal/min) and the liquid meal (2.55 kcal/min), indicating comparable nutrient delivery to the small intestine and further corroborating that meal texture did not meaningfully alter gastric processing under the controlled conditions of our study. Our findings are consistent with this concept and suggest that, at least for the motility parameters quantified here, caloric load may exert a stronger influence than physical texture on measurable postprandial motor dynamics.

The persistent localization of the capsule in the fundus provides additional mechanistic insights. In all participants, the capsule remained in the proximal stomach and was not observed to enter the antrum or undergo gastric emptying. This observation is consistent with established fed-state physiology, in which non-digestible solid particles are selectively retained until the reappearance of phase III of the migrating motor complex (MMC). A combination of telemetric capsule and scintigraphic study by Coupe et al. demonstrated that solid indigestible markers administered with meals may remain in the stomach for several hours and are typically emptied only after restoration of interdigestive motor activity (Coupe et al., 1991). Similar findings have been reported in pharmaceutical imaging studies by Weitschies et al., showing prolonged and highly variable gastric residence of non-disintegrating dosage forms under fed conditions (Weitschies et al., 2010; Weitschies et al., 2005). Within this framework, the absence of capsule emptying in our study does not indicate impaired motility but rather reflects normal spatial and functional compartmentalization of the fed stomach. Postprandially, the fundus primarily serves as a reservoir with limited propulsive activity, whereas effective trituration and pyloric transport occur predominantly in the distal stomach. In addition, it should be considered that intragastric distribution and transit may depend on the characteristics of the dosage form. While disintegrating formulations such as tablets may be transferred more rapidly towards the antrum after ingestion, non-disintegrating solid forms, such as the capsule investigated in this study may exhibit prolonged retention in the proximal stomach (Graham et al., 1990). Taken together, the lack of observed antral entry of the capsule therefore suggests that size, density, non-digestibility, fed-state motor suppression of the MMC as well as the supine position of the participants are decisive determinants of its behavior, independent of subtle differences in measurable contraction metrics between meal consistencies.

From a pharmaceutical perspective, these findings might have direct implications for enteric-coated dosage forms. Our observations reinforce the necessity for robust acid resistance of enteric coatings, particularly when administration occurs in the fed state, where gastric residence times may substantially exceed commonly anticipated durations, particularly during daytime conditions. Ensuring coating integrity under prolonged low-pH exposure is therefore critical to prevent premature drug release, particularly because the fed-state stomach exhibits prolonged elevations in intragastric pH compared with the fasted state, which can modify the dissolution behavior of pH-dependent coatings such as those used for enteric protection (Fadda et al., 2022).

Also, a few limitations need consideration. The sample size was limited, and subtle differences in coordination patterns may not have been detectable. Furthermore, while meals were isocaloric, minor differences in nutrient composition and intragastric homogenization cannot be entirely excluded. The imaging duration (∼ 82 min) was chosen as a compromise between capturing the relevant postprandial phase and maintaining sufficient gastric filling for reliable rtMRI-based motility analysis. As gastric emptying progresses, reduced luminal content leads to diminished intragastric contrast, limiting the assessment of peristaltic activity (Neubauer et al., 2026). Consequently, later events such as antral entry or gastric emptying of the capsule could not be observed. While a longer observation period focusing on capsule transit might have provided additional insights, this was beyond the primary scope of the study.

Beyond providing physiological insights, the datasets generated in this study also enhance the methodological robustness and versatility of our AI-driven imaging framework. By retraining our artificial intelligence models *Volyntra* and *Motiqva* with these measurements, we established two robust AI tools capable of accurately segmenting and analyzing gastric emptying and peristaltic activity across four distinct gastric contents: water, pineapple juice (Neubauer et al., 2026), a light solid meal, and Fresubin Energy. This enhances the versatility of our imaging platform and lays the foundation for future studies investigating gastric motility and oral dosage form behavior under a wide range of nutritional conditions.

Taken together, our findings indicate that, under isocaloric conditions, measurable postprandial antral motility is largely independent of meal consistency. However, these findings should be interpreted within the specific context of the controlled isocaloric conditions applied in this study. Gastric motility responses are known to vary substantially with meal composition, including caloric load, viscosity, fibre content, and volume, which may also influence outcomes in standardized pharmacokinetic or food-effect study settings. The rapid emptying of co-administered water and the persistent fundic retention of a non-disintegrating capsule illustrate established fed-state physiology. From a practical perspective, these results suggest that standardized liquid nutrition such as Fresubin Energy may provide a reproducible and clinically relevant option for investigating gastric motility, emptying or dosage form behavior under fed conditions in translational or mechanistic studies. Importantly, this does not imply equivalence to the high-fat FDA standard breakfast, which is intentionally designed to maximize potential food effects in regulatory pharmacokinetic testing. Rather, a standardized liquid meal may be particularly suitable for controlled physiological investigations or clinical scenarios where extreme worst-case nutritional conditions are not the primary focus.

Given that oral dosage forms in many PK studies are administered after a waiting period of approximately 30 min, typically beyond the lag phase of solid meals, the choice of a liquid test meal under isocaloric conditions may not substantially alter the motility environment encountered by the dosage form in such settings.

## 5 Conclusion

Under isocaloric conditions, postprandial antral motility appeared to be largely independent of meal consistency, with contraction frequencies around 3 cpm and propagation velocities of circa 2 mm/s consistent with published physiological ranges. The rapid water emptying along the *Magenstrasse* and persistent fundic retention of the capsule reinforce established fed-state mechanisms. From a methodological and translational perspective, these findings suggest that a standardized liquid meal may offer a practical and physiologically controlled alternative for mechanistic studies and clinical research settings. Furthermore, the integration of retrained AI models provides robust tools for quantitative analysis of gastric emptying and peristalsis across multiple meal types, thereby facilitating future investigations of oral dosage form behavior and disease- or medication-induced alterations in gastric motility.

## CRediT authorship contribution statement

**Lydia Neubauer:** Writing – original draft, Methodology, Investigation, Conceptualization. **Simon Bartels:** Investigation. **Fiona Mankertz:** Resources, Investigation. **Dirk Voit:** Software, Resources. **Jens Frahm:** Software, Resources. **Werner Weitschies:** Writing – review & editing, Supervision, Conceptualization. **Linus Großmann:** Writing – review & editing, Supervision, Project administration, Methodology, Investigation, Conceptualization.

## Funding

The University of Greifswald received funding from the German Research Foundation (DFG, INST 292/155–1 FUGG). This study was also supported by Health.AI Pomerania (grant INT0100008).

## Declaration of competing interest

All authors declare that they have no known competing financial interests or personal relationships that could have appeared to influence the work in this publication.

## Data Availability

Data will be made available on valid request due to data protection and ethics limitation.
